# Novel Highly Pathogenic Avian Influenza A(H5N6) Viruses in the Netherlands

**DOI:** 10.3201/eid2407.180635

**Published:** 2018-07

**Authors:** Yifei Xu

**Affiliations:** University of Oxford, Oxford, UK

**Keywords:** highly pathogenic avian influenza, H5N6, clade 2.3.4.4 H5Nx, reassortment, influenza, influenza virus, viruses, respiratory infections, zoonoses, Netherlands, the Netherlands

**To the Editor:** In their recent article, Beerens et al. (*1*) reported detection of a novel clade 2.3.4.4 group B highly pathogenic avian influenza (HPAI) H5N6 subtype virus in wild birds and poultry in the Netherlands in December 2017. This novel virus is a reassortant of the HPAI H5N8 subtype virus with polymerase basic 2 (PB2) and neuraminidase genes from the Eurasian gene pool. This study highlighted concerns over the recent emergence and spread of these viruses. H5N6 subtype viruses have been identified in multiple Eurasian regions and have shown diverse genotypes since the beginning of 2017. Clade 2.3.4.4 group B H5N6 subtype viruses were reported to have affected migratory birds and poultry in South Korea in December 2017 (*2*). The viruses from South Korea have PB2 and polymerase acidic genes distinct from those of viruses from the Netherlands. H5N6 subtype viruses were also detected in Greece, Japan, and Taiwan in 2017. Viruses from these regions have the same genotype, but PB2, polymerase acidic, and neuraminidase genes differ from those in the viruses from the Netherlands. 

Clade 2.3.4.4 group A H5N8 subtype viruses emerged in East Asia in early 2014 and subsequently spread, along migratory bird routes, to Europe and North America by the end of 2014. H5N8 subtype and reassortant viruses, including H5N1 and H5N2 subtypes (*3*), resulted in the deaths of 7.5 million turkeys and 42.1 million chickens in the United States alone. Since June 2016, these H5N8 subtype viruses have circulated in Europe and reassorted with local low pathogenicity avian influenza viruses. Group B H5N8 subtype viruses caused the long-lasting second wave of outbreaks in wild birds and poultry in 30 countries in Europe, including the Netherlands, Belgium, Luxembourg, and the United Kingdom (*4*). It is hard to predict whether novel clade 2.3.4.4 group B H5N6 subtype viruses might trigger a similar new wave of massive HPAI H5Nx outbreaks in wild and domestic birds and even cause infections among poultry workers. However, more surveillance data are needed to investigate the genetic diversity and evolution of these viruses and how widely they are circulating in wild birds. 
